# Multidrug-Resistant CTX-M-(15, 9, 2)- and KPC-2-Producing *Enterobacter hormaechei* and *Enterobacter asburiae* Isolates Possessed a Set of Acquired Heavy Metal Tolerance Genes Including a Chromosomal *sil* Operon (for Acquired Silver Resistance)

**DOI:** 10.3389/fmicb.2018.00539

**Published:** 2018-03-23

**Authors:** Leonardo N. Andrade, Thiago E. S. Siqueira, Roberto Martinez, Ana Lucia C. Darini

**Affiliations:** ^1^Faculdade de Ciencias Farmaceuticas de Ribeirao Preto, Universidade de Sao Paulo, Ribeirao Preto, Brazil; ^2^Faculdade de Medicina de Ribeirao Preto, Universidade de Sao Paulo, Ribeirao Preto, Brazil

**Keywords:** silver, copper, ESBL, carbapenemase, *Enterobacter cloacae* complex species

## Abstract

Bacterial resistance to antibiotics is concern in healthcare-associated infections. On the other hand, bacterial tolerance to other antimicrobials, like heavy metals, has been neglected and underestimated in hospital pathogens. Silver has long been used as an antimicrobial agent and it seems to be an important indicator of heavy metal tolerance. To explore this perspective, we searched for the presence of acquired silver resistance genes (*sil* operon: *silE, silS, silR, silC, silF, silB, silA*, and *silP*) and acquired extended-spectrum cephalosporin and carbapenem resistance genes (*bla*_CTX−M_ and *bla*_KPC_) in *Enterobacter cloacae* Complex (EcC) (*n* = 27) and *Enterobacter aerogenes* (*n* = 8) isolated from inpatients at a general hospital. Moreover, the genetic background of the *silA* (silver-efflux pump) and the presence of other acquired heavy metal tolerance genes, *pcoD* (copper-efflux pump), *arsB* (arsenite-efflux pump), *terF* (tellurite resistance protein), and *merA* (mercuric reductase) were also investigated. Outstandingly, 21/27 (78%) EcC isolates harbored *silA* gene located in the chromosome. Complete *sil* operon was found in 19/21 *silA*-positive EcC isolates. Interestingly, 8/20 (40%) *E. hormaechei* and 5/6 (83%) *E. asburiae* co-harbored *silA/pcoD* genes and *bla*_CTX−M−(15,2,or9)_ and/or *bla*_KPC−2_ genes. Frequent occurrences of *arsB, terF*, and *merA* genes were detected, especially in *silA/pcoD*-positive, multidrug-resistant (MDR) and/or CTX-M-producing isolates. Our study showed co-presence of antibiotic and heavy metal tolerance genes in MDR EcC isolates. In our viewpoint, there are few studies regarding to bacterial heavy metal tolerance and we call attention for more investigations and discussion about this issue in different hospital pathogens.

*Enterobacter* species have been found to be among the top five Gram-negative bacilli that cause hospital infections (Mezzatesta et al., 2012; Davin-Regli and Pages, 2015). Given the increasing identification of carbapenem-resistant and third-generation cephalosporin-resistant *Enterobacter* isolates, these bacteria are recognized as priority one pathogens (i.e., a critical genus within the family *Enterobacteriaceae*), according to the World Health Organization (http://www.who.int).

Antibiotic resistance has been widely studied in order to control healthcare-associated infections. On the other hand, tolerance to other antimicrobials (non-antibiotic compounds) has been neglected and underestimated in hospital pathogens. Heavy metals are used to treat superficial bacterial infections and to prevent microbial contamination and control proliferation (e.g., silver and copper), and they are also considered food and/or feed contaminants (e.g., mercury and arsenic) and industrial pollution contaminants (e.g., tellurium; Lemire et al., 2013; Hobman and Crossman, 2015).

Silver is the main non-antibiotic substance that has long been used as an antimicrobial agent and it seems to be an important indicator of heavy metal tolerance. The silver-based substances include silver nitrate and, more recently, silver nanoparticles (nanosilver) have been developed. Bacterial metal homeostasis does not require silver to be metabolized. On the contrary, silver is highly toxic to bacteria, causing cell damage via several mechanisms (Maillard and Hartemann, 2012; Barillo and Marx, 2014; Chandrangsu et al., 2017). Acquired silver resistance was reported to involve derepression of the SilCFBA efflux transporter as a consequence of a mutation in an exogenous (acquired) *sil* operon gene and additional expression of a periplasmic silver-sequestration protein (Randall et al., 2015), contributing to reduced intracellular accumulation and activity of silver.

*sil* operon genes (*silE, silS, silR silC, silF, silB, silA, ORF105*, and *silP*) code for apparently structural proteins and for a putative two-component regulatory circuit. SilA (efflux pump), SilB (accessory membrane fusion protein), and SilC (outer membrane porin) correspond to the resistance-nodulation-division (RND)-type efflux transporter SilCBA (efflux system); SilE and SilF are periplasmic silver-sequestration proteins; SilP is a putative P-type ATPase transporter; SilR and SilS comprise a putative two-component regulatory circuit; and ORF105 has an amino acid sequence that is homologous to a putative copper chaperone known as CopG, thus it codes for the hypothetical protein SilG, as previously described (Randall et al., 2015). Some studies showed that Sil proteins (e.g., SilE, SilP, and SilCBA efflux system) are also involved in copper tolerance, mainly under anaerobic conditions (Silver, 2003; Kim et al., 2011; Mourão et al., 2015, 2016).

Moreover, other acquired genes have been associated with heavy metal tolerance, such as *pcoD* (copper efflux pump), *arsB* (arsenite efflux pump), *terF* (tellurite resistance protein), and *merA* (mercuric reductase) (Hobman and Crossman, 2015; Mourão et al., 2016). Thus, the genetic determinants and mechanisms of heavy metal tolerance have been characterized and linked to enzymic detoxification or efflux of metals.

Considering the few molecular and epidemiological data on the distribution of *sil* operon genes and the genetic background of silver resistance, and, more generally, the few data on acquired heavy metal tolerance genes in hospital pathogens, we searched for the presence of *sil* operon genes and beta-lactamase-encoding genes in *Enterobacter* species isolated from inpatients at a general hospital. Moreover, the genetic background of the *silA* gene and the presence of other heavy metal tolerance genes were also investigated. In addition, *Enterobacter cloacae* Complex (EcC) species identification and a bacterial population structure analysis were performed.

We investigated 27 EcC and eight *Enterobacter aerogenes* isolates (both antibiotic-susceptible and -resistant isolates) recovered from non-repeated inpatients, involving different types of clinical samples and different wards at a university hospital in Brazil (December 2014–April 2015). Clonality was assessed by carrying out genomic DNA digestion with *Xba*I followed by pulsed-field gel electrophoresis (PFGE), and the results were analyzed using the Tenover criteria (Tenover, 2001) to determine the pulsotypes. EcC species assignment was performed by *hsp60* partial gene sequencing (Hoffmann and Roggenkamp, 2003). Multidrug-resistance (MDR) phenotype was defined as non-susceptibility to at least one agent from three or more antibiotic categories (Magiorakos et al., 2012). *sil* operon genes (*silE, silS, silR, silC, silF, silB, silA*, and *silP*) as well as other genes for heavy metal tolerance (*pcoD, arsB, terF*, and *merA*) were investigated by PCR (Woods et al., 2009; Kremer and Hoffmann, 2012; Randall et al., 2015; Mourão et al., 2016). The localization of the *sil*A gene was investigated by carrying out genomic DNA digestion with *S1* and *I-CEU-I*-nuclease followed by PFGE, southern blot and hybridization (Ferreira et al., 2014). Genes coding for ESBLs (*bla*_CTX−M_, _TEM, SHV_) and carbapenemases (*bla*_KPC, IMP, VIM, NDM, OXA−48_) were also searched for using PCR and sequencing (Woodford et al., 2006; Bogaerts et al., 2013).

Among the 27 EcC isolates, 20 (74%; 20 pulsotypes) were identified as *Enterobacter hormaechei*, six (22%; 5 pulsotypes) as *Enterobacter asburiae*, and just one (4%) as *E. cloacae* (Table 1). Outstandingly, 21/27 (78%) EcC isolates harbored *silA* gene. Notably, *silA* and *pcoD* genes were found in 14/20 (70%) *E. hormaechei* and all *E. asburiae* isolates. Only a single *E. cloacae* isolate (which was *silA* positive) had no *pcoD* gene. Moreover, some EcC isolates also carried *arsB* (*n* = 11) and/or *terF* (*n* = 10) and/or *merA* (*n* = 6) genes; all of them were *silA/pcoD* positive, except for one *E. hormaechei* (E04) isolate that was *silA/pcoD* negative and carried only *terF* and *merA* genes. The *E. aerogenes* isolates did not possess the heavy metal resistance genes investigated (Table 1).

**Table 1:** Heavy metal resistance genes, *bla* genes, and antibiotic-resistance phenotypes of the *Enterobacter* spp. isolates studied.

**Species**	**Isolate^#^**	**Heavy metal resistance genes**	***bla* genes**	Antibiotic resistance profile^*^	**Resistance phenotype**
*E. asburiae*	E06	*silA, pcoD, arsB*	–	–	–
*E. asburiae*	E11	*silA, pcoD, arsB, terF*	CTX-M-9	TZP, CTX, CAZ	–
*E. asburiae*	E20	*silA, pcoD, arsB*	CTX-M-9	TZP, CTX, CAZ, IPM	MDR
*E. asburiae*	E17	*silA, pcoD, arsB, terF, merA*	CTX-M-9	TZP, CTX, CAZ, FEP, IPM, MER, GEN	MDR
*E. asburiae*	E46	*silA, pcoD, arsB, terF, merA*	CTX-M-9, KPC-2	TZP, CTX, CAZ, FEP, ERT, IMP, MER, NAL, SXT, GEN, NIT	MDR
*E. asburiae*	E43	*silA, pcoD*	CTX-M-15	CTX, STX, GEN, NIT	MDR
*E. hormaechei*	E21	*silA, pcoD, terF*	CTX-M-15	TZP, CTX, CAZ, FEP, CIP, TGC	MDR
*E. hormaechei*	E22	*silA, pcoD, arsB, terF, merA*	CTX-M-15	TZP, CTX, CAZ, FEP, ERT, CIP, GEN, TGC	MDR
*E. hormaechei*	E42	*silA, pcoD, arsB, terF, merA*	CTX-M-15	TZP, CTX, FEP, NAL, NOR, CIP, STX, GEN, NIT	MDR
*E. hormaechei*	E44	*silA, pcoD, arsB, terF, merA*	CTX-M-15	CTX, STX, GEN, NIT	MDR
*E. hormaechei*	E40	*silA, pcoD, arsB, terF*	CTX-M-15	TZP, CTX, ERT, MER, NAL, NOR, CIP, STX, GEN, NIT	MDR
*E. hormaechei*	E50	*silA, pcoD, arsB, terF*	CTX-M-15	CTX, CAZ, FEP, GEN	-
*E. hormaechei*	E49	*silA, pcoD, arsB, terF*	CTX-M-2	TZP, CTX, CAZ, FEP, CIP, GEN	MDR
*E. hormaechei*	E31	*silA, pcoD*	KPC-2	TZP, CTX, CAZ, FEP, ERT, IPM, MER, NAL, NOR, CIP, TGC, NIT	MDR
*E. hormaechei*	E08	*silA, pcoD*	–	–	–
*E. hormaechei*	E09	*silA, pcoD*	–	–	–
*E. hormaechei*	E12	*silA, pcoD*	–	–	–
*E. hormaechei*	E13	*silA, pcoD*	–	–	–
*E. hormaechei*	E47	*silA, pcoD*	–	–	–
*E. hormaechei*	E19	*silA, pcoD, merA*	–	TZP, CTX, CAZ	–
*E. hormaechei*	E07	–	–	TZP, CTX, CAZ	–
*E. hormaechei*	E04	*terF, merA*	–	NIT	–
*E. hormaechei*	E03	–	–	NAL, NOR, CIP, STX, NIT	–
*E. hormaechei*	E10	–	–	NIT	–
*E. hormaechei*	E15	–	–	-	–
*E. hormaechei*	E51	–	–	-	–
*E. cloacae*	E48	*silA*	–	-	–
*E. aerogenes*	E01	–	–	NIT	–
*E. aerogenes*	E02	–	–	NIT	–
*E. aerogenes*	E05	–	–	TZP, NIT	–
*E. aerogenes*	E14	–	–	-	–
*E. aerogenes*	E23	–	–	NIT	–
*E. aerogenes*	E29	–	–	-	–
*E. aerogenes*	E39	–	–	-	–
*E. aerogenes*	E41	–	–	NIT	–

# *E. asburiae E11 and E17 isolates belonged to same pulsotype. The other EcC isolates and E. aerogenes isolates corresponded to different pulsotypes; E. hormaechei E07 and E19 isolates displayed cefotaxime and ceftazidime resistance due to overproducing AmpC (ESBL negative) (data not shown)*.

* *Piperacillin-tazobactam (TZP), cefotaxime (CTX), ceftazidime (CAZ), cefepime (FEP), ertapenem (ERT), imipenem (IPM), meropenem (MER), ertapenem (ERT), gentamicin (GEN), nitrofurantoin (NIT), nalidixic acid (NAL), norfloxacin (NOR), ciprofloxacin (CIP), trimethoprim-sulfamethoxazole (SXT), tigecycline (TGC)*.

*MDR, multidrug-resistance (Magiorakos et al., 2012). For the determination of the MDR phenotype, antibiotic intrinsic resistance (data not shown) and antibiotic therapeutic concentrations achieved only in urine (e.g., in the case of nitrofurantoin) were excluded; Enterobacter cloacae Complex (EcC) species and Enterobacter aerogenes are intrinsically resistant to ampicillin, ampicillin-sulbactam, amoxicillin-clavulanate, first-generation cephalosporin (cefazolin and cephalothin), second-generation cephalosporin (cefuroxime), and cephamycins (cefoxitin and cefotetan)*.

Complete *sil* operon genes were found in 19/21 *silA*-positive EcC isolates. In the other two isolates, one, *E. hormaechei* (E21), did not possess the *silB* gene, and the other, *E. cloacae* (E48), only possessed the *silA* gene. Curiously, *silC* and *silR* were detected in all the *E. aerogenes* isolates (all of which were *silA* negative). All *silA*-positive EcC isolates possessed the *silA* gene in the chromosome, although most bacteria studied harbored plasmids, as visualized by *S1*-PFGE.

Interestingly, 8/20 (40%) *E. hormaechei* and 5/6 (83%) *E. asburiae* co-harbored *silA/pcoD* genes and *bla*_CTX−M−(15, 2, or 9)_ and/or *bla*_KPC−2_ genes. Regarding the MDR phenotype, 7/20 (35%) *E. hormaechei* and 4/6 (67%) *E. asburiae* were MDR, amounting to a total of 11/26 EcC isolates that were *silA* positive and CTX-M and/or *Klebsiella pneumoniae* Carbapenemase (KPC) producers. Nevertheless, the single *E. cloacae* and the *E. aerogenes* isolates neither possessed acquired *bla* genes nor the MDR phenotype (Table 1).

*E. hormaechei* and *E. asburiae* were the most frequently identified EcC species and they appear to be common EcC species isolated from human infections, representing a concern because there are few data on these species (Mezzatesta et al., 2012). The high diversity of pulsotypes among the *E. hormaechei* and *E. asburiae* isolates as well as the high occurrence of the *silA* gene in these species suggest non-clonal spread and long-term silver resistance among these species at the hospital studied.

EcC species appear to be important *sil*-carrying bacteria compared to other Gram-negative bacilli (Woods et al., 2009; Sütterlin et al., 2012). A silver-resistant *E. cloacae* strain was previously detected with several *sil* operon genes (*silE, silP*, and *silS*) but no *silA* gene (potentially due to it not being searched for; Sütterlin et al., 2012; Finley et al., 2015). The silver-resistance phenotype in EcC isolates might also occur due to derepression of the endogenous *cus* operon and loss of porins, as characterized previously (Randall et al., 2015).

The *sil* operon has been reported to be inducible in the presence of silver (Randall et al., 2015), which also explains the non-direct phenotypic resistance among isolates carrying *sil* genes. Thus, the inducible expression of silver resistance could contribute to the selection for silver-resistant pathogens, mainly among bacteria carrying the complete *sil* operon and overexpressing the SilCFBA efflux transporter (Randall et al., 2015).

The chromosomal *silA* location indicates that *silA* provides an advantageous trait, as this genetic determinant of silver resistance was detected in multiple clones of *E. hormaechei* and *E. asburiae* and in a single *E. cloacae* isolate. Chromosomal integration of acquired antimicrobial-resistance genes, commonly plasmid-mediated, has been reported in laboratory and *in silico* studies; the chromosomal integration is likely explained by genetic uptake and chromosomal recombination/integration. In addition, plasmids harboring *silA* (and/or *pcoD*) genes have also been detected, mainly in *Salmonella* (Ferreira et al., 2014; Fang et al., 2016; Mourão et al., 2016).

The *pcoD* gene (along with the *silA* gene) was very common in *E. hormaechei* and *E. asburiae* isolates and it also seems to be a frequently acquired heavy metal resistance gene in these EcC species. Copper is essential for bacterial metabolism at normal cellular concentrations. Nevertheless, at toxic concentrations, copper causes damage in bacterial cells by several different mechanisms of action (Lemire et al., 2013; Hobman and Crossman, 2015; Staehlin et al., 2016; Chandrangsu et al., 2017). In addition to the obvious contribution to copper tolerance, *pcoD* (and *silA*) genes have been related to the emergence of specific clinically relevant MDR *Salmonella* serotypes/clones, highlighting the relevance of copper tolerance in anaerobic conditions (Mourão et al., 2016). In addition, the *pcoD* operon and copper efflux has been shown to be associated with bacterial survival in amoebae, and copper-resistance determinants have been reported to be located in a “copper pathogenicity island,” contributing to the selection of copper-resistant bacteria. The *pco* operon has frequently been found to be encoded adjacent to the *sil* operon, contributing to heavy metal resistance co-selection (Woods et al., 2009; Hao et al., 2015, 2016; Pal et al., 2015).

Interestingly, the acquired heavy metal resistance genes *arsB, terF*, and *merA* were also detected in the *E. hormaechei* and *E. asburiae* isolates, particularly in the *silA/pcoD*-positive, MDR and/or CTX-M-producing isolates. Several interesting associations involving theses heavy metal resistance genes have been demonstrated. For example, the *arsB* gene is more related to *Salmonella enterica* serovar Kentucky than other serovars isolated from poultry and the *ars, sil*, and *pco* operons are co-carried on epidemic plasmids (e.g., IncFII_K1_) in MDR *K. pneumoniae* clones (Joerger et al., 2010; Sandegren et al., 2012; Chen et al., 2013; Chen and Rosen, 2014). In addition, tellurite resistance (*terW* gene, a *ter* operon gene) is commonly reported in community hypervirulent *K. pneumoniae* clonal groups (Taylor, 1999; Passet and Brisse, 2015), and *mer* operon genes are associated with many Tn21-like transposons that usually co-harbor antibiotic-resistance genes (Boyd and Barkay, 2012). Thus, arsenic, tellurium, mercury, as well as copper tolerance and/or other advantageous characteristics, need further investigation in *Enterobacter* species and other hospital pathogens.

The selection of antibiotic-resistant bacteria has been amplified by co-resistance, that is, the presence of different resistance mechanisms (encoded by mutated or acquired genes) affecting different antimicrobial classes (Cantón and Ruiz-Garbajosa, 2011; Wong et al., 2014; Kumar et al., 2016). We demonstrated that the CTX-M- and/or KPC-producing EcC isolates studied here (most of which were MDR bacteria) possessed a larger set of heavy metal tolerance genes (all of which were *silA/pcoD* positive and most of which were also *arsB, terF*, and *merA* positive) than the non-acquired beta-lactamase producers, indicating co-resistance involving antibiotics and heavy metals and suggesting co-selection of silver/copper- and acquired cephalosporin/carbapenem-resistance genes. Likewise, the *silA/pcoD*-negative results seem to be more associated with *E. aerogenes* than EcC species (Table 1). There was a close association between the *bla*_CTX−M_ gene and the *silA* (and *pcoD*) genes among the isolates studied here, and this finding (silver-resistance genes and/or silver-resistance phenotype) was previously reported for clinical *Escherichia coli* isolates (Sütterlin et al., 2012, 2014; Deus et al., 2017). Moreover, there have been reports of silver-resistant (and/or *silA*-positive) isolates associated with silver minimal inhibitory concentrations that could impact the management of non-invasive bacterial infections (such as those found among burn patients; Finley et al., 2015; Deus et al., 2017).

Notably, MDR bacteria are a worldwide problem and have caused antibiotic failure, so new alternative antibiotics and treatment approaches have been studied (Dizaj et al., 2014; de Oliveira et al., 2017; Wang et al., 2017). The combined action of nanosilver and antibiotics has been shown to have additive or synergistic antibacterial action, including the ability to restore the bactericidal activity of inactive antibiotics against MDR *Enterobacteriaceae* and *Pseudomonas aeruginosa* (Panácek et al., 2015, 2016; Salomoni et al., 2017). However, the versatility and practicality of nanosilver treatment for bacteria that are co-resistant to antibiotics and silver should be investigated in future studies, including comparison with soluble silver salts. For this purpose, it is essential to know the epidemiology of the genetic determinants of silver resistance and to understand the molecular mechanisms of silver resistance in different hospital pathogens. Our study contributes to this understanding, and it characterized acquired silver-resistance genes in clinically important bacterial species that were isolated from multiple inpatients, clinical samples, and wards at a general hospital.

Considering the increasing use of silver in medical devices (e.g., catheters and dressings) and consumer household products (e.g., antiperspirants and clothes), and also based on the dramatic increase in reports of silver-resistant bacteria, silver resistance should be a concern beyond the silver resistance seen in isolates infecting burn patients (Lemire et al., 2013; Marx and Barillo, 2014; Hobman and Crossman, 2015).

In summary, our study indicates the common occurrence of acquired *sil* operon genes and the *pcoD* gene exclusively in the EcC species, which display high clonal diversity. The study highlights the presence of the *silA* gene in the chromosome, indicating that it gives rise to an advantageous and adaptive trait. Frequent occurrences of *arsB, terF*, and *merA* genes were also detected, especially in *silA/pcoD*-positive, MDR and/or CTX-M-producing isolates. The co-presence of acquired silver/copper-resistance (*silA/pcoD*) and cephalosporin/carbapenem-resistance (*bla*_CTX−M_ and *bla*_KPC−2_) genes in the MDR EcC isolates was noteworthy.

Due to absence of surveillance and laboratory testing, bacterial heavy metal resistance can silently boost the antibiotic resistance contributing to selection and spreading of MDR bacteria. In our viewpoint, there are few studies regarding to bacterial heavy metal tolerance and we call attention to more investigations and discussion about this issue, toward detection and monitoring of this neglected and underestimated problem in different hospital pathogens.

## Author contributions

LA was responsible for the study, and performed all the molecular characterizations of the bacteria and the antibiotic- and heavy metal resistance genes. TS and RM carried out the microbiological investigation of the bacterial isolates. LA and AD coordinated the research and wrote the manuscript.

### Conflict of interest statement

The authors declare that the research was conducted in the absence of any commercial or financial relationships that could be construed as a potential conflict of interest.
